# Procedural characteristics of pulmonary vein isolation with high-power short-duration setting compared to conventional setting

**DOI:** 10.1186/s12872-022-02459-2

**Published:** 2022-01-24

**Authors:** Naoko Hijioka, Takashi Kaneshiro, Takeshi Nehashi, Kazuaki Amami, Minoru Nodera, Shinya Yamada, Masashi Kamioka, Takafumi Ishida, Yasuchika Takeishi

**Affiliations:** 1 Department of Cardiovascular Medicine, Fukushima Medical University, 1 Hikarigaoka, Fukushima, 960-1295 Japan; 2 Department of Arrhythmia and Cardiac Pacing, Fukushima Medical University, Fukushima, Japan

**Keywords:** High-power short-duration ablation, Atrial fibrillation, Pulmonary vein isolation, First pass isolation, Dormant conduction

## Abstract

**Purpose:**

The purpose of this study was to investigate the safety and efficacy of high-power short-duration (HP-SD) ablation compared to conventional ablation in patients with atrial fibrillation (AF).

**Methods:**

We enrolled consecutive 158 drug-refractory symptomatic AF patients (119 males, mean age 63 ± 10 years) who had undergone first radiofrequency pulmonary vein isolation (PVI). PVI was performed using the conventional setting (20–35 W) in 73 patients (Conventional group) and using the HP-SD setting (45–50 W) in 85 patients (HP-SD group). The rate of first pass isolation, remaining gaps after circumferential ablation, dormant conduction, and the radiofrequency application time in each pulmonary vein (PV) were compared between the groups.

**Results:**

The first pass isolation ratio was significantly higher in the HP-SD group than in the Conventional group (81% vs. 65%, *P* = 0.027) in the right PV, but did not differ in the left PV. The remaining gaps were fewer in the right superior PV (4% vs. 21%, *P* = 0.001) and left inferior PV (1% vs. 8%, *P* = 0.032) areas, and the radiofrequency application time in each PV was shorter (right PV, 12.0 ± 8.9 min vs. 34.0 ± 31.7 min, *P* < 0.001; left PV, 10.6 ± 3.6 min vs. 25.7 ± 22.3 min, *P* < 0.001) in the HP-SD group than in the Conventional group.

**Conclusion:**

The use of the HP-SD setting might contribute to improve the first pass isolation rate and to shorten the radiofrequency application time in each PV.

**Supplementary Information:**

The online version contains supplementary material available at 10.1186/s12872-022-02459-2.

## Introduction

Atrial fibrillation (AF) has been recognized as the most common sustained arrhythmia and is associated with both cardiovascular mortality and substantial morbidity. [1–3] Pulmonary vein isolation (PVI) is an established therapy because of its efficacy. [4, 5] However, it is still a major concern to decrease AF recurrence after PVI. Therefore, the development of an innovative approach to decrease AF recurrence after PVI is important. Recently, high-power short-duration (HP-SD) ablation has been introduced as a new PVI method, and its efficacy and safety have been validated in previous studies. [6–8] The HP-SD setting might shorten the conductive heating phase and lead to the reduction of collateral tissue damage. However, lesion characteristics during the HP-SD ablation procedure have not yet been evaluated. The aim of the present study was to investigate procedural characteristics of PVI using the HP-SD setting compared to the conventional setting.

## Methods

### Study population

**Fig. 1:**
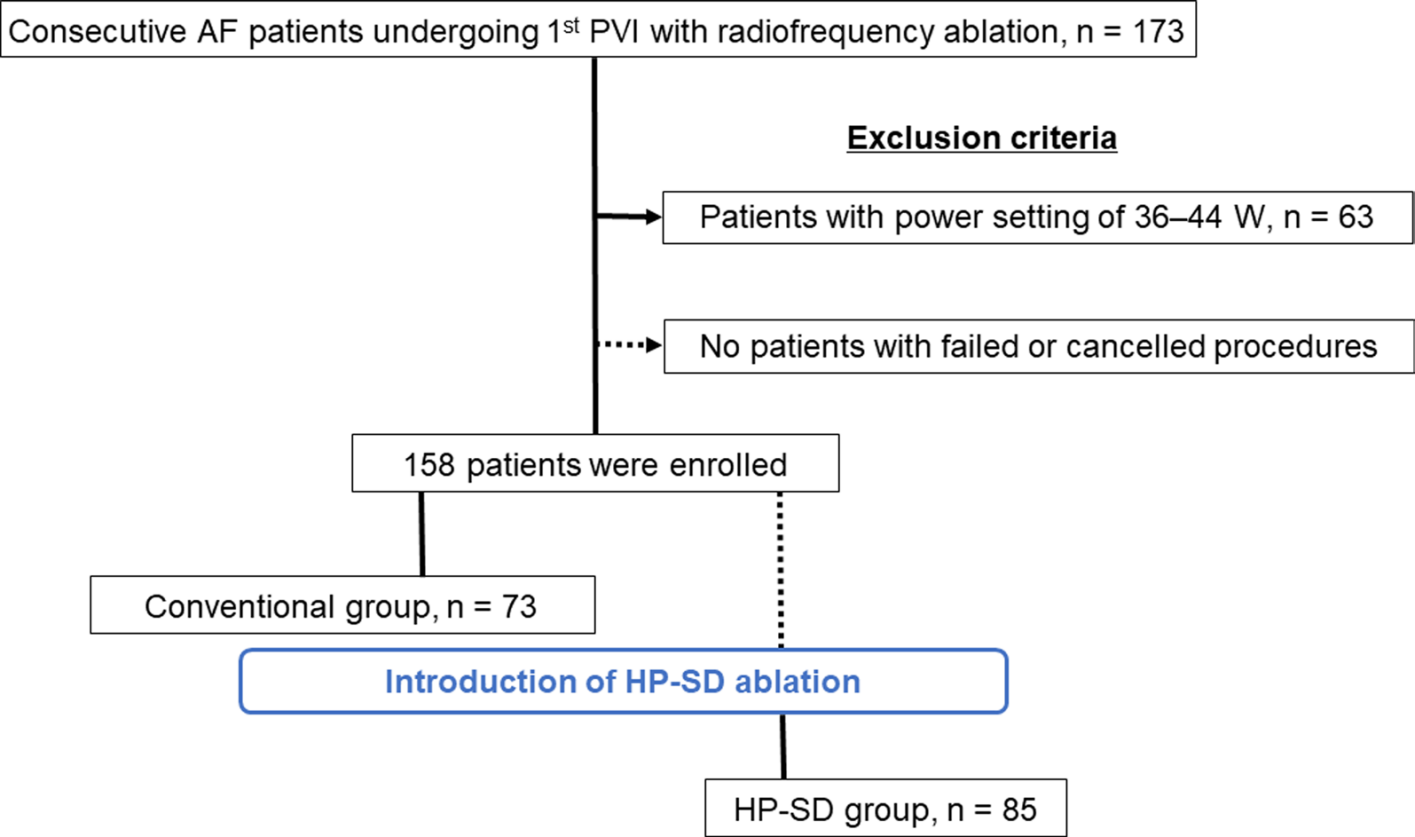
Patient flow diagram. After the exclusion criteria, the study patients were divided into two groups based on the used power setting. AF, atrial fibrillation; HP-SD, high-power short-duration; PVI, pulmonary vein isolation

We retrospectively enrolled 173 consecutive AF patients who had successfully undergone first PVI for symptomatic drug-refractory AF at our hospital between January 2018 and October 2019, during when we replaced AF ablation with the HP-SD setting (Fig. 1). Echocardiography and computed tomography revealed no structural heart disease in any patient. The patients were divided into two groups based on the used radiofrequency power output setting. We analyzed the first 73 patients who underwent ablation in a conventional setting with a radiofrequency power of 20–35 W before the introduction of HP-SD setting (Conventional group), and the following 85 patients who underwent HP-SD ablation with a radiofrequency power of 45–50 W (HP-SD group). Fifteen patients were excluded because they had undergone the procedure at a radiofrequency power of 36–44 W. Finally, 158 patients (119 males, mean age 63 ± 10 years, 99 paroxysmal AF) were enrolled in the study. Written informed consent was obtained from all study subjects, and the study was approved by the ethics committee of Fukushima Medical University.

### Catheter ablation of atrial fibrillation

Our institution has 1 electrophysiological laboratory and 4 electrophysiologists. We had performed 275 catheter ablation procedures per year in average during study period, the 10% of these procedures for ventricular tachyarrhythmias and the remaining 90% for atrial arrhythmias such as AF, supraventricular tachycardia and atrial tachycardia. The detailed procedural protocol of PVI using radiofrequency catheter ablation at our hospital was as previously described. [9] Each patient underwent an electrophysiologic study and first radiofrequency catheter ablation. Anticoagulative drugs were continued or minimally discontinued during perioperative days at the individual operator’s discretion. [10, 11] Anti-arrhythmic drugs were discontinued for at least five half-lives. PVI was performed under sedation with intravenous dexmedetomidine and fentanyl. A 10-polar catheter was positioned at the His bundle area via the right femoral vein to record a His potential. A 20-polar 3-site (CS, lateral right atrium and SVC) mapping catheter (BeeAT, Japan-Life-Line, Tokyo, Japan) was inserted via the right subclavian vein. Proximal 4-polar electrodes were located at the superior vena cava (SVC), middle 8-polar electrodes at the right atrium, and distal 10-polar at the coronary sinus (CS). Twelve-lead surface electrocardiograms (ECGs) and intracardiac electrograms were recorded simultaneously by digital multichannel systems (Prucka CardioLab®, General Electrical Healthcare, Milwaukee, WI, USA and RMC-5000, Nihon Kohden Corp. Tokyo, Japan), filtered at 30–400 Hz for bipolar and 0.05–400 Hz for unipolar electrograms.

All PVI was performed using a 3-dimensional electro-anatomical mapping system (CARTO system, Biosense Webster, Diamond Bar, CA, USA). In the Conventional group, an 8-Fr irrigation catheter with a 3.5-mm distal electrode and real-time contact force (CF) monitoring (ThermoCool SmartTouch, Biosense Webster) or an 8-Fr surround flow irrigation catheter with a 3.5-mm distal electrode and real-time CF monitoring, which has a 56-hole porous tip for surround flow irrigation (ThermoCool SmartTouch SF, Biosense Webster) was used for radiofrequency energy delivery. Radiofrequency energy was delivered at 20–35 W using a point-by-point or dragging technique at the individual operator’s discretion. In the HP-SD group, ThermoCool SmartTouch SF was used for radiofrequency energy delivery. Radiofrequency energy was delivered at 45–50 W using a point-by-point technique and ablation index (AI) module (Biosense Webster). AI is a novel lesion quantity parameter that incorporates CF, time, and power in a weighted formula, and has been shown to accurately estimate lesion depth in canine studies. [12, 13] The duration of radiofrequency application was determined by AI value in the present study. The targeted AI was set at 380–400 in the left posterior PV, and at 400–430 at other sites in the left PV (LPV) and right PV (RPV). The targeted CF were 10–25 g in the Conventional group and 5–20 g in the HP-SD group. Force time integral (FTI) was defined as the total CF integrated over the duration of radiofrequency current delivery at each ablation point. After completion of PVI, intravenous adenosine (20 mg) was administered to check for dormant LA-PV conduction in the right PVs during sinus rhythm and in the left PVs during distal CS pacing with continuous administration of isoproterenol (1.0–3.0 mg/min).

### Analysis of procedural parameters during PVI

**Fig. 2:**
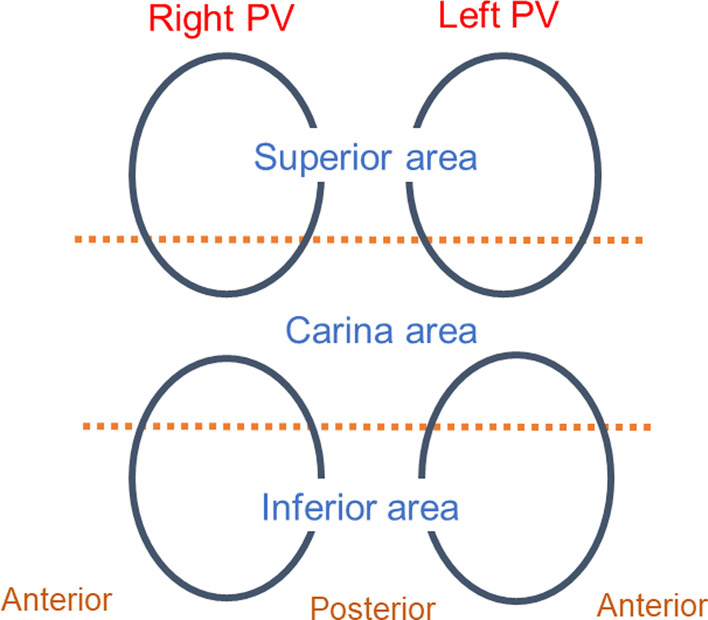
Schematic diagram of separation of each pulmonary vein. PV was divided in six areas: the superior, carina, and inferior areas of each side of the PV. PV, pulmonary vein

PV was divided in six areas: the superior, carina, and inferior areas of each side of the PV. The prevalence of first pass isolation, remaining gaps between the left atrium (LA) and PV after circumferential ablation and dormant conduction after completion of PVI were also investigated in each of the six areas (Fig. 2). Radiofrequency application time in each side of the PVI and total procedure time were also calculated.

### Follow-up

Follow-up was performed at 1, 3 and 6 months after the procedure, and every 3 months thereafter. At each visit, 12-lead ECG, and 24-h Holter monitoring were performed. Recurrence was defined as documentation of atrial tachycardia, or AF lasting > 30 s recorded by 12-lead ECG or 24-h Holter monitoring. AAD were continued for 3 months after PVI and discontinued thereafter. The long-term efficacy was assessed clinically on the basis of the clinical symptoms, surface 12-lead ECG, and 24-h Holter monitoring.

### Statistical analysis

A Chi-square test was used for comparisons of categorical variables, which are expressed as numbers and percentages. Continuous variables are presented as mean ± standard deviation, and were compared using Student’s t-test. All analyses were performed using SPSS for Windows, version 25.0 (SPSS Inc., Chicago, IL, USA), and all statistical tests were two-sided. A P-value of < 0.05 was considered statistically significant (All data analyzed during this study are included in the Additional file 1).

## Results

### Patient characteristics

The patient characteristics are summarized in Table 1. There were no significant differences in sex, age, type of AF, body mass index, BNP, estimated glomerular filtration rate, left atrial dimension, left atrial volume index, or left ventricular ejection fraction between the two groups (Table 2).

**Table 1:** Baseline characteristics of the study subjects

	Total (n = 158)	Conventional (n = 73)	HP-SD (n = 85)	*P*
Sex, male, n (%)	119 (75%)	55 (75%)	64 (75%)	0.994
Age, years	62.9 ± 10.1	61.6 ± 11.1	64.1 ± 9.1	0.136
Type of AF, paroxysmal	99 (62%)	46 (63%)	53 (62%)	0.932
Body mass index	24.6 ± 3.9	24.6 ± 3.7	24.5 ± 4.1	0.956
BNP, pg/mL	89.1 ± 125.4	85.4 ± 107.3	92.1 ± 138.9	0.742
eGFR, mL/min/1.73 m^2^	63.9 ± 38.1	61.2 ± 12.3	66.2 ± 50.7	0.421
LAD, mm	40.8 ± 6.5	40.7 ± 6.6	40.9 ± 6.5	0.891
LAVI, mL/m^2^	43.7 ± 16.4	42.4 ± 16.8	44.8 ± 16.0	0.415
LVEF, %	61.2 ± 9.7	61.4 ± 8.4	61.0 ± 10.7	0.793

AF, atrial fibrillation; BNP, brain natriuretic peptide; eGFR, estimated glomerular filtration rate; HP-SD, high-power short-duration; LAD, left atrial dimension; LAVI, left atrial volume index; LVEF, left ventricular ejection fraction

**Table 2:** Procedural results of right pulmonary vein isolation

	Conventional (n = 73)	HP-SD (n = 85)	*P*
First pass isolation	48 (65%)	69 (81%)	0.027
*Remaining gap*			
Right superior PV	16 (21%)	4 (4%)	0.001
Right carina	11 (15%)	7 (8%)	0.178
Right inferior PV	5 (6%)	5 (5%)	0.803
Dormant conduction	5 (6%)	1 (1%)	0.062
Ablation time, min	34.0 ± 31.7	12.0 ± 8.9	< 0.001

HP-SD, high-power short-duration; PV, pulmonary vein

### Comparison of procedural characteristics between the Conventional and HP-SD settings

**Fig. 3:**
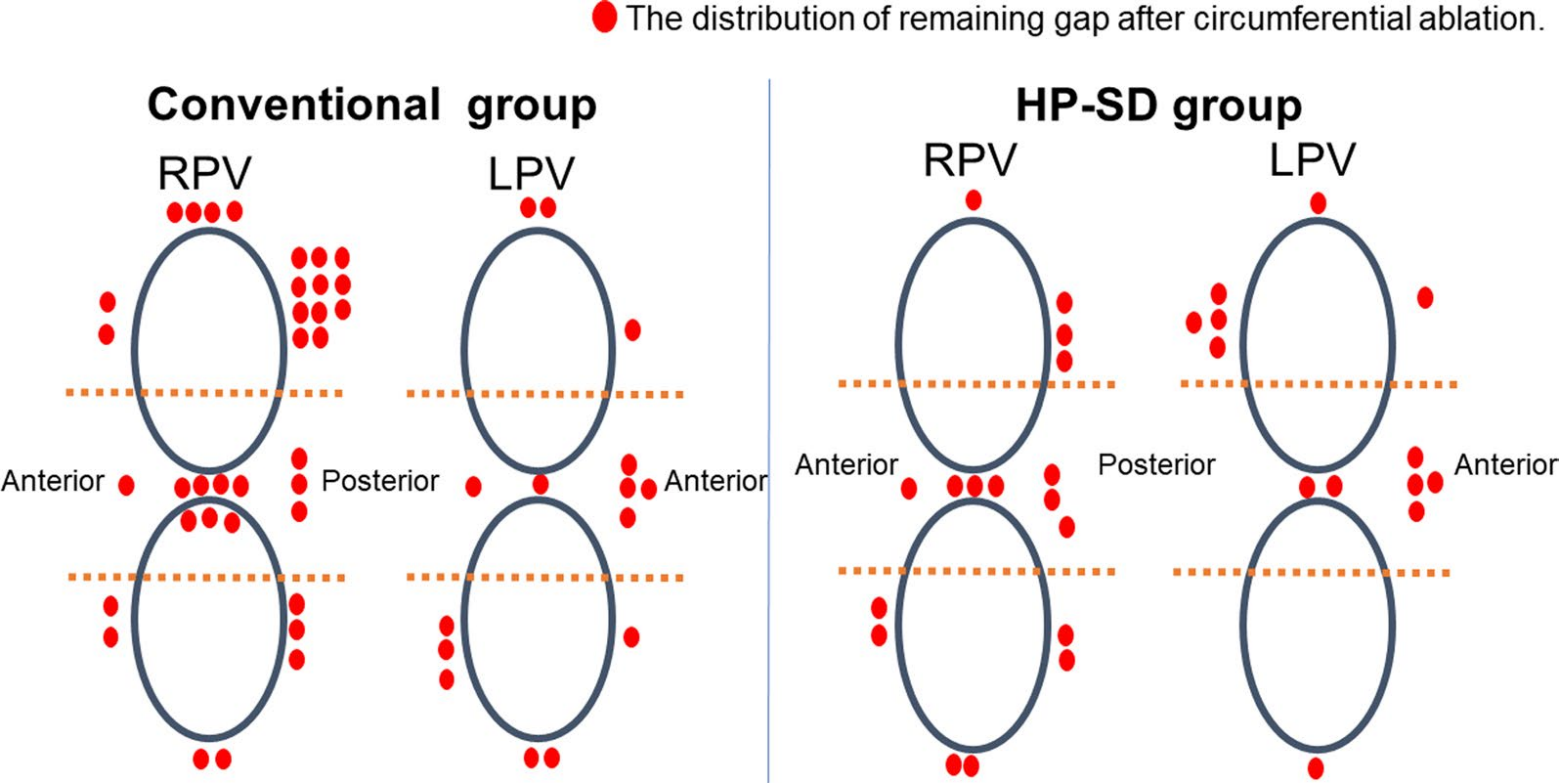
Distribution of the remaining gaps after circumferential ablation in each PV. The remaining gaps after circumferential ablation were fewer in the right superior PV and the left inferior PV areas in the HP-SD group compared to the Conventional group. HP-SD, high-power short-duration; LPV, left pulmonary vein; RPV, right pulmonary vein

**Fig. 4:**
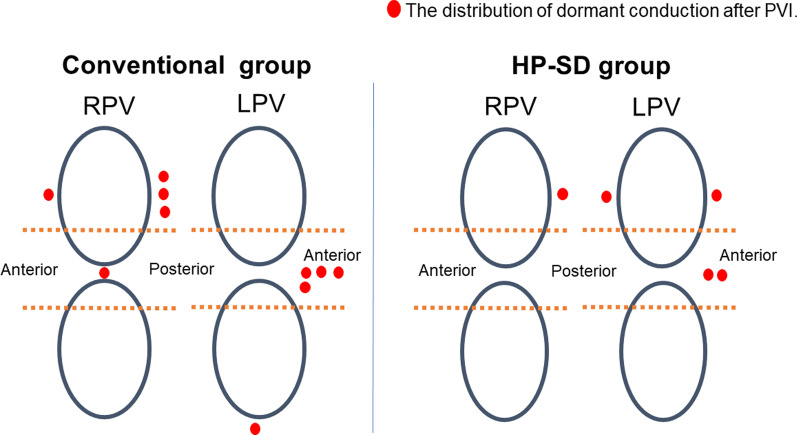
Distribution of the dormant conduction after PVI in each PV. The dormant conduction tended to be fewer in the HP-SD group than in the Conventional group in the RPV. HP-SD, high-power short-duration; LPV, left pulmonary vein; RPV, right pulmonary vein; PVI, pulmonary vein isolation

In the RPV, first pass isolation was achieved more frequently in the HP-SD group than in the Conventional group (81% vs. 65%, *P* = 0.027) (Table 3). However, the rate of first pass isolation in the LPV did not differ between the two groups (83% vs. 80%, *P* = 0.657) (Table 3). The remaining gaps after circumferential ablation were fewer in the right superior PV (4% vs. 21%, *P* = 0.001) and the left inferior PV (1% vs. 8%, *P* = 0.032) areas in the HP-SD group compared to the Conventional group (Fig. 3). The dormant conduction tended to be fewer in the HP-SD group than in the Conventional group in the RPV (1% vs. 6%, *P* = 0.062) (Fig. 4). In addition, radiofrequency application time in each side of the PV was shorter in the HP-SD group than in the Conventional group (RPV,

**Table 3:** Procedural results of left pulmonary vein isolation

	Conventional (n = 73)	HP-SD (n = 85)	*P*
First pass isolation	59 (80%)	71 (83%)	0.657
*Remaining gap*			
Left superior PV	3 (4%)	6 (7%)	0.425
Left carina	6 (8%)	7 (8%)	0.997
Left inferior PV	6 (8%)	1 (1%)	0.032
Dormant conduction	4 (5%)	4 (4%)	0.823
Ablation time, min	25.7 ± 22.3	10.6 ± 3.6	< 0.001

HP-SD, high-power short-duration; PV, pulmonary vein

12.0 ± 8.9 min vs. 34.0 ± 31.7 min, *P* < 0.001; LPV, 10.6 ± 3.6 min vs. 25.7 ± 22.3 min, *P* < 0.001). The total procedure time was also shorter in the HP-SD group than in the Conventional groups (171.8 ± 40.8 min vs. 193.1 ± 45.3 min, *P* = 0.002).

### Clinical outcome after PVI

**Fig. 5:**
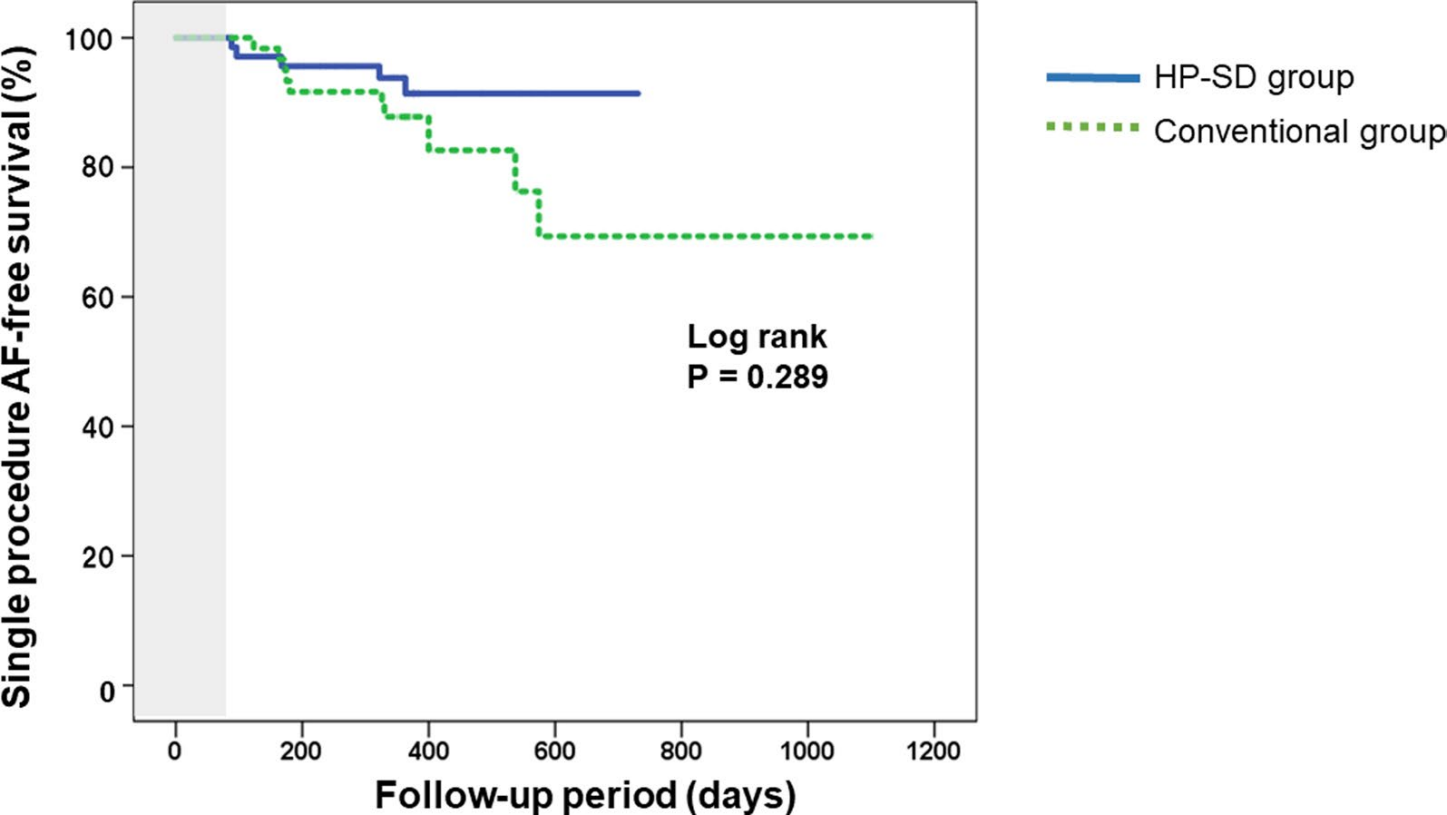
The Kaplan–Meier time-to-event curves for recurrence after pulmonary vein isolation. The patients in the Conventional group exhibited a higher recurrence rate than those in the HP-SD group. AF, atrial fibrillation; HP-SD, high-power short-duration

During a follow-up period of 753 days, recurrence of atrial arrhythmia was documented in 15 patients (11%). With respect to the comparison of ablation outcome between the two groups, atrial arrythmia recurrence seemed to be more frequent in the Conventional group (16.3% vs. 7.1%, *P* = 0.083). The Kaplan–Meier time-to-event curves for recurrence showed that the recurrence rate in the Conventional group seemed to be higher than that in the HP-SD group (Log rank; *P* = 0.289) (Fig. 5). Regarding major ablation-related complications during PVI, pericardial effusion was observed in two patients in the Conventional group but in no patients in the HP-SD group. The other complications were never occurred in both groups.

## Discussion

The major findings of the present study were that the HP-SD setting was able to improve the rate of first pass isolation, reduce the remaining gap after circumferential ablation and the radiofrequency application time as well as the total procedure time. Moreover, this setting might reduce the dormant conduction after circumferential ablation and the rate of atrial tachyarrhythmia recurrence.

### Previous findings about HP-SD ablation

Recently, several studies have revealed the efficacy of HP-SD ablation. Okamatsu, et al. showed that HP-SD ablation could shorten the time to complete circumferential PVI and improve the rate of first pass isolation. [6] In addition, an extremely low rate of complications was demonstrated by using HP-SD ablation. [6, 8]

### Procedural characteristics of HP-SD ablation

Our data also revealed that HP-SD ablation could shorten radiofrequency application time and total procedure time. AI was calculated with ablation time, radiofrequency power, catheter stability and CF. [12, 13] To achieve the targeted AI, the needed radiofrequency application time would be shorter when using HP-SD setting compared to conventional setting. Moreover, the reduced remaining gap might shorten the procedure time by avoiding the additional radiofrequency application. Thus, it was acceptable that both radiofrequency application time and procedure time were shortened in the HP-SD group.

The results of the present study showed the trend of lower prevalence of remaining gaps after circumferential PVI in the HP-SD group. Mulder et al. revealed that local LA wall thickness was associated with acute PV reconnection after PVI. [14] In addition, our previous report showed that the presence of hypertension induced heterogeneous LA wall hypertrophic change and LA remodeling. [15] Therefore, it might be possible that the LA wall thickness might affect the existence of remaining gaps after circumferential PVI.

### Clinical implication

The safety of AI guided ablation has already been validated in some reports. [7, 12, 13] In the present study, the major complication during PVI was pericardial effusion, which occurred in two patients of the Conventional group but in no patients of the HP-SD group; and no other complications occurred in either group. No major complication in the HP-SD group in the present study suggests that the safety of the HP-SD setting. Moreover, the advantage of HP-SD setting has been shown to prevent collateral tissue damage by shortening the resistive heating phase. [8, 16] Especially, the prevention of esophageal complication is very important because esophageal mucosal injury has the potential to advance to left atrial-esophagus fistula, which is a fatal complication associated with PVI. [17, 18] As we had reported the advantage of HP-SD setting in preventing esophageal complications, we should take advantage of this setting in regard to safety. [19, 20]

### Study limitations

There are several limitations to the present study. First, it was a single center observational study; therefore, further evaluation in a multicenter, randomized control study is necessary. Second, the follow-up period was relatively short in the HP-SD group. A large-scale study with a longer follow up period is needed to investigate further. Third, the present study showed the favorable trend of the long-term efficacy when using HP-SD setting. However, there was a lack of data about the AF duration of each patient in both groups, and the impact on the long-term efficacy in the present study might be limited.

## Conclusions

The results of the present study indicate that the HP-SD setting might contribute to improve the rate of first pass isolation and reduce the radiofrequency application time as well as total procedure time. Moreover, the HP-SD setting might contribute to reduce the recurrence of atrial tachyarrhythmia after PVI using HP-SD setting.

## Supplementary Information

**Additional file 1.** The results of patient characteristics, procedural details of ablation and follow-up data after ablation procedures.

## Data Availability

All data analyzed during this study are included in the supplementary information files.
